# A Prospective Incidence Study on Sexually Transmitted Infections and HIV in Men Who Have Sex With Men, With or Without Use of Pre-exposure Prophylaxis

**DOI:** 10.1093/ofid/ofag205

**Published:** 2026-04-11

**Authors:** Klaus Jansen, Trevor A Crowell, Patrick F Ottensmeyer, Allahna L Esber, Gyde Steffen, Heiko K Jessen, Christiane Cordes, Stefan Scholten, Stephan Schneeweiss, Norbert H Brockmeyer, Christoph D Spinner, Markus Bickel, Stefan Esser, Axel Baumgarten, Albrecht Stoehr, Clara Lehmann, Jörg J Vehreschild, Tsedal Mebrahtu, Julie Dorsey-Spitz, Alexandra Knorr, Anna-Lena Brillen, Jernej Pusnik, Carsten Tiemann, Nelson L Michael, Merlin L Robb, Hendrik Streeck

**Affiliations:** Department for Infectious Disease Epidemiology, Robert Koch Institute, Berlin, Germany; U.S. Military HIV Research Program, CIDR, Walter Reed Army Institute of Research, Silver Spring, Maryland, USA; Henry M. Jackson Foundation for the Advancement of Military Medicine, Bethesda, Maryland, USA; Institute of Virology, Medical Faculty, University Bonn, Bonn, Germany; German Center for Infection Research (DZIF), Partner Site Bonn-Cologne, Bonn, Germany; U.S. Military HIV Research Program, CIDR, Walter Reed Army Institute of Research, Silver Spring, Maryland, USA; Henry M. Jackson Foundation for the Advancement of Military Medicine, Bethesda, Maryland, USA; Department for Infectious Disease Epidemiology, Robert Koch Institute, Berlin, Germany; Private practice, Jessen+Jessen, Berlin, Germany; Infektionsmedizin Berlin-Friedrichshain, Berlin, Germany; Private practice, Hohenstaufenring, Cologne, Germany; Private practice, Hohenstaufenring, Cologne, Germany; WIR–Walk in Ruhr, Center for Sexual Health and Medicine, Bochum, Germany; Interdisciplinary Immunological Outpatient Clinic, Center for Sexual Health and Medicine, Department of Dermatology, Venereology and Allergology, Ruhr University Bochum, Bochum, Germany; TUM School of Medicine and Health, Department of Clinical Medicine–Clinical Department for Internal Medicine II, University Medical Center, Technical University of Munich, Munich, Germany; Infektiologikum Frankfurt, Frankfurt, Germany; Institute for the Research on HIV and AIDS-Associated Diseases Essen, University Duisburg-Essen, Essen, Germany; HPSTD Clinic, Department of Dermatology, University Hospital Essen, University Duisburg-Essen, Essen, Germany; zfi– Zentrum für Infektiologie Berlin Prenzlauer Berg GmbH, Berlin, Germany; Institute for Interdisciplinary Medicine, Hamburg, Germany; Department I for Internal Medicine, Faculty of Medicine at University Hospital Cologne, Cologne, Germany; German Center for Infection Research (DZIF), Partner Site Bonn-Cologne, Bonn, Germany; Department I for Internal Medicine, Faculty of Medicine at University Hospital Cologne, Cologne, Germany; U.S. Military HIV Research Program, CIDR, Walter Reed Army Institute of Research, Silver Spring, Maryland, USA; Henry M. Jackson Foundation for the Advancement of Military Medicine, Bethesda, Maryland, USA; U.S. Military HIV Research Program, CIDR, Walter Reed Army Institute of Research, Silver Spring, Maryland, USA; Henry M. Jackson Foundation for the Advancement of Military Medicine, Bethesda, Maryland, USA; Institute for the Research on HIV and AIDS-Associated Diseases Essen, University Duisburg-Essen, Essen, Germany; Institute for the Research on HIV and AIDS-Associated Diseases Essen, University Duisburg-Essen, Essen, Germany; Institute of Virology, Medical Faculty, University Bonn, Bonn, Germany; German Center for Infection Research (DZIF), Partner Site Bonn-Cologne, Bonn, Germany; Laboratory Krone, Bad Salzuflen, Germany; Walter Reed Army Institute of Research, Silver Spring, Maryland, USA; U.S. Military HIV Research Program, CIDR, Walter Reed Army Institute of Research, Silver Spring, Maryland, USA; Henry M. Jackson Foundation for the Advancement of Military Medicine, Bethesda, Maryland, USA; Institute of Virology, Medical Faculty, University Bonn, Bonn, Germany; German Center for Infection Research (DZIF), Partner Site Bonn-Cologne, Bonn, Germany; Institute for the Research on HIV and AIDS-Associated Diseases Essen, University Duisburg-Essen, Essen, Germany; HPSTD Clinic, Department of Dermatology, University Hospital Essen, University Duisburg-Essen, Essen, Germany

## Abstract

**Background:**

While pre-exposure prophylaxis (PrEP) can prevent HIV acquisition, associated behavioral changes may increase risk of acquiring other sexually transmitted infections (STIs).

**Methods:**

The prospective multicenter BRAHMS study enrolled men who were HIV negative, had sex with men, and were aged 18 to 55 years and reported an increased risk to acquire STI. At 3-month intervals for up to 12 months, participants answered questionnaires and underwent site-specific screening for *Chlamydia trachomatis* (CT), *Mycoplasma genitalium* (MG), *Neisseria gonorrhoeae* (NG), and syphilis. We computed 3-month incidence rates and rate ratios, and we employed multilevel mixed effects logistic regression to determine odds ratios (ORs) and 95% CIs for factors associated with any incident STI.

**Results:**

The mean age of 1017 participants was 33 years, 54.7% used PrEP at enrollment, and 83.7% reported PrEP use overall. Any STI was diagnosed in 71.8% (CT, 40.4%; MG, 38.5%; NG, 36.0%; syphilis, 10.1%; HIV, 0.5%). PrEP users exhibited a higher prevalence for anorectal infections with CT (35.3% vs 20.5%, *P* < .05), MG (31.4% vs 23.5%, *P* < .05), and NG (26.1% vs 16.9%, *P* < .05). CT incidence decreased from 46.8 cases per 100 person-years to 35.7 (−23.7%, *P* < .05), and NG incidence decreased from 41.6 cases per 100 person-years to 30.7 (−26.2%, *P* < .05). MG and syphilis incidence remained stable. Factors independently associated with STI incidence were as follows: symptoms (OR, 1.85; 95% CI, 1.46–2.34), condomless anal sex with >5 casual partners (OR, 1.79; 95% CI, 1.49–2.14), recreational drug use (OR, 1.76; 95% CI, 1.46–2.12), being born abroad (OR, 1.49; 95% CI, 1.19–1.87), PrEP use (OR, 1.29; 95% CI, 1.05–1.58), and having a moderately or largely increased self-perceived risk of HIV (OR, 1.26 [95% CI, 1.03–1.53]; OR, 1.33 [95% CI, 1.02–1.74], respectively).

**Conclusions:**

PrEP use was associated with increased STI risk in our cohort. CT and NG incidence decreased in a structured test-and-treat setting.

## Teaser

Pre-exposure prophylaxis (PrEP) users exhibited higher prevalence for anorectal infections with chlamydia, mycoplasma, and gonorrhea than non-PrEP users. Reduction of chlamydia and gonorrhea incidence in a test-and-treat setting supports the integration of risk-adapted sexually transmitted infection screening based on assessment of the individual risk of infection acquisition among clients within PrEP services; it also highlights the importance of addressing sexual health broadly, including drug use and the challenges of managing asymptomatic infections.

Pre-exposure prophylaxis (PrEP) has demonstrated remarkable efficacy in preventing HIV transmission [1]. However, its effectiveness could inadvertently lead to shifts in behavior, including reduced condom use or an increase in the number of sexual partners [2, 3]. These behavioral changes may increase the risk of contracting other sexually transmitted infections (STIs), such as *Chlamydia trachomatis* (CT), *Mycoplasma genitalium* (MG), *Neisseria gonorrhoeae* (NG), or *Treponema pallidum* (TP; syphilis) [4, 5].

STIs represent a significant global health challenge, and men who have sex with men (MSM) face a disproportionate burden [6]. Clinical guidelines recommend PrEP for MSM who have a history of STIs or multiple sexual partners [7, 8], reflecting the increased risk of STIs in this group [5, 9]. Consequently, most guidelines advise regular screening for various STIs while undergoing PrEP [7, 8]. Such frequent STI screening could lead to a higher proportion of diagnosed and treated STIs. This approach has the potential to curb STI transmission within the PrEP user population [3, 10, 11].

In Germany, PrEP was approved in 2016. MSM with increased sexual risk behavior and/or recent STI are eligible for PrEP [7]. German-Austrian guidelines recommend TP testing for PrEP users every 3 months; CT and NG could be tested every 3 to 6 months and should be tested yearly. Since September 2019, German compulsory health insurance covers the costs of PrEP and all corresponding tests (HIV, STI, creatinine), and PrEP uptake increased to about 40 000 PrEP users currently [12].

Research systematically comparing STI incidence between PrEP users and non-PrEP users is limited, particularly when it comes to aligning with screening intervals recommended in clinical PrEP guidelines. Studies examining STI incidence among MSM using PrEP have reported high rates, ranging from 55.4 to 91.9 per 100 person-years (PY) [4, 5, 9, 11, 13]. These variations can be attributed to differences in the pathogens studied, specific research methodologies, and the characteristics of the populations studied.

The overall advantage of frequent STI screening in populations with a higher STI incidence is under debate. Frequently treating asymptomatic STIs may raise concerns about the overuse of antibiotics, potentially contributing to antibiotic resistance [14] and disrupting the individual microbiome, with emphasis on asymptomatic MG infections, for which current guidelines do not recommend treatment [15–18]. In addition, the anatomic locations to screen are under debate, especially regarding pharyngeal infections, which might clear naturally [3, 19–21].

Hence, we conducted a comprehensive assessment of STI prevalence and incidence as the primary study aim within a large multisite cohort of MSM who met criteria for PrEP eligibility to depict STI dynamics over time in a structured test-and-treat setting. Our study placed emphasis on examining various pathogens, anatomic locations, and the impact of PrEP usage.

## METHODS

### Study Procedures

The study setup and protocol of the BRAHMS study (ClinicalTrials.gov NCT03884816) have been reported [22]. In summary, the BRAHMS study was a prospective multicenter study that enrolled participants without HIV who identified as male (at birth, chosen, or intersexual), were aged 18 to 55 years, and reported risk to acquire HIV and other STIs based on either of the following in the past 24 weeks: (1) diagnosis of syphilis, acute hepatitis C, or rectal infection with MG, CT, or NG or (2) condomless anal intercourse with ≥2 male partners with HIV or unknown status. The study was conducted at 10 sites in 7 major German cities (Berlin, Bochum, Cologne, Essen, Frankfurt, Hamburg, Munich) between June 2018 and March 2021.

At intervals of 3 months for up to 12 months (hereafter, visits V0, V3, V6, V9, and V12), participants underwent counseling sessions focused on PrEP education and were given the opportunity to undergo HIV testing. During these study visits, participants received risk reduction counseling and were provided with condoms.

### Behavioral and Clinical Data Collection

Upon enrollment in the study, participants were queried about their sociodemographic characteristics and sexual behaviors. During each subsequent visit, participants were interviewed regarding their sexual behaviors since the last visit. Furthermore, we gathered comprehensive clinical data, which encompassed information on participants’ active use of PrEP at the time of their visit and any clinical symptoms potentially consistent with an STI on the date of the visit (eg, urethral or rectal burning, discharge or bleeding, sore throat or difficulties swallowing, genital or rectal ulcers, and others).

### Diagnosis of STIs

At each visit, blood was collected to test for syphilis. The serologic examination was carried out in accordance with European guidelines [23] using a syphilis-specific polyvalent screening test (Syphilis TP chemiluminescence microparticle immunoassay; Abbott), which in positive cases was followed by diagnostic confirmation consisting of a TP particle agglutination assay (Fujirebio), 2 fluorescence antibody absorption tests (IgG-FTA-abs and 19S-IgM-FTA-abs; Zeus/Euroimmun), and a rapid plasma reagin test (Becton Dickinson). Disease activity and the need for treatment were determined by detecting *Treponema*-specific IgM antibodies and/or non–*Treponema*-specific lipoid antibodies. A differentiation between initial *Treponema* infections and reinfections was made, taking into account all available anamnestic information or a comparison of results with findings from previous examinations. On this basis, syphilis infection was defined as active contagious infection requiring antibiotic treatment in the study.

Voided urine, anal, and oropharyngeal swabs were collected at each visit and individually tested as follows: for CT and NG, by the nucleic acid amplification test Aptima Combo 2; for MG, by the Aptima *Mycoplasma genitalium* Assay; and for *Trichomonas vaginalis*, by the Aptima *Trichomonas vaginalis* Assay (all assays by Hologic). All testing was performed according to manufacturer instructions. In case of a positive STI test result, treatment was offered according to current clinical guidelines.

### Statistical Analysis

We described the study population by calculating frequencies and proportions for dichotomous and categorical variables and the median with IQR for continuous variables. Population characteristics were compared by Pearson χ^2^ test and Mann-Whitney *U* test as appropriate.

We calculated 3-month incidence rates and incidence rate ratios for comparison. We used multilevel mixed effects logistic regression to calculate odds ratios (ORs) and 95% CIs for factors potentially associated with any incident STI. For this purpose, we used a cross-sectional approach including each visit of each participant as a single observation to enable evaluation of changing individual behaviors in terms of PrEP use, sexual behavior, substance use, and other evaluated characteristics. By the multilevel mixed effect modeling, we were able to take possible participant-specific associations into account. Data points with missing values were not included in multivariable analyses. The level of significance was set to .05 for all conducted tests.

The BRAHMS study was paused due to the first COVID-19–related lockdown in Germany between 24 March and 22 July 2020. After this lockdown, a small proportion of final study visits (V12) were conducted (2.7% of all study visits, 13.7% of all V12 visits). Therefore, we introduced a variable specifying if a visit was conducted before or after this lockdown to allow for analysis of and adjustment for a potential independent impact of the COVID-19 pandemic on STI incidence.

Analyses were performed in Stata 17.0 (StataCorp).

### Ethical Considerations

The study was approved by institutional review boards of the Walter Reed Army Institute of Research, the University Duisburg-Essen, and all collaborating institutions (17-7598-BO). All participants provided written informed consent prior to any study procedures.

## RESULTS

### Participant Characteristics

Our study included 1017 participants at enrollment (V0), with 1009 participating in V3 (99.2%), 1000 (98.3%) in V6, 985 (96.9%) in V9, and 969 (95.3%) in V12, resulting in a 4.7% rate of loss to follow-up over the entire study period.

**Figure 1.**
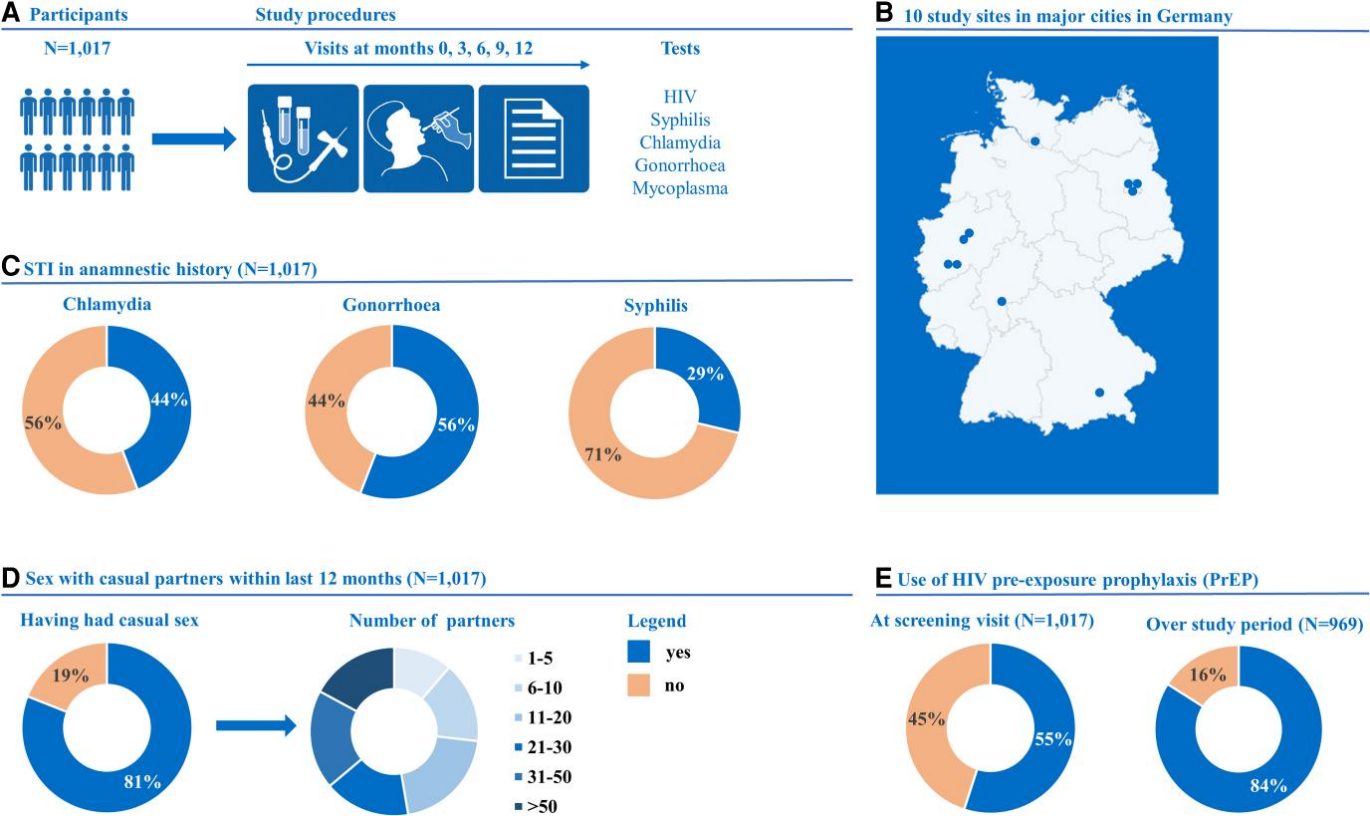
Study flow and select participant characteristics at enrollment into the BRAHMS cohort. STI, sexually transmitted infection.

Overall, 54.7% (n = 556) reported PrEP usage at enrollment (Figure 1, Table 1). Over the course of the study, PrEP usage increased to 73.3% (n = 710) at V12. PrEP use was reported at least once during the entire study duration by 83.7% (n = 851).

**Table 1.** Select Participant Characteristics by Use of HIV PrEP at Enrollment Into the BRAHMS Cohort

	All Participants (n = 1017)		Not Used PrEP (n = 461)		Used PrEP (n = 556)		
	No.	%	No.	%	No.	%	*P* Value
Age, y							**<.05**
18–29	329	32.4	191	41.4	138	24.8	
30–39	473	46.5	199	43.2	274	49.3	
40–49	183	18.0	57	12.4	126	22.7	
50–55	32	3.1	14	3.0	18	3.2	
Gender identity							.39
Cisgender man	1008	99.1	457	99.1	551	99.1	
Nonbinary	5	0.5	1	0.2	0	0	
Transgender man	1	0.1	1	0.2	0	0	
Transgender woman	1	0.1	2	0.4	3	0.5	
Gender queer	2	0.2	0	0	2	0.4	
Sexual orientation							.29
Gay or homosexual	936	92.1	420	91.1	516	92.8	
Bisexual	48	4.7	27	5.9	21	3.8	
Heterosexual, other,^a^ unknown	33	3.2	14	3.0	19	3.4	
Place of birth							.061
Germany	690	67.8	326	70.7	364	65.5	
Other country	320	31.5	131	28.4	189	34.0	
Missing	7	0.7	4	0.9	3	0.5	
City							**<.05**
Berlin	383	37.7	166	36.0	217	39.0	
Bochum	144	14.2	62	13.4	82	14.7	
Cologne	162	15.9	72	15.6	90	16.2	
Essen	74	7.3	45	9.8	29	5.2	
Frankfurt	99	9.7	38	8.2	61	11.0	
Hamburg	30	2.9	10	2.2	20	3.6	
Munich	125	12.3	68	14.8	57	10.3	
Education							**<.05**
Less than secondary school	31	3.1	14	3.0	17	3.2	
Secondary school	451	44.3	230	49.9	221	39.7	
Undergraduate degree	171	16.8	79	17.2	92	16.5	
Master or doctorate	364	35.8	138	29.9	226	40.6	
No. of casual sex partners within last 12 mo							**<.05**
No casual partner	54	5.3	28	6.1	26	4.7	
1–5	93	9.1	59	12.8	34	6.1	
6–10	128	12.6	65	14.1	63	11.3	
11–20	169	16.6	76	16.5	93	16.7	
21–30	136	13.4	75	16.3	61	11.0	
31–40	94	9.2	39	8.5	55	9.9	
41–50	63	6.2	20	4.3	43	7.7	
>50	141	13.9	43	9.3	96	17.6	
Missing	139	13.7	56	12.1	83	14.9	
Condomless anal intercourse with casual sex partners within last 12 months	766	75.3	338	73.3	428	77.0	**<.05**
Lifetime drug use							
Illicit	667	65.6	307	66.6	360	64.7	.54
Recreational ^a^	559	55.0	255	55.3	304	54.7	.84
STI in medical history							
*Chlamydia trachomatis*	448	44.1	174	37.7	274	49.3	**<.05**
*Neisseria gonorrhoeae*	569	55.9	232	50.3	337	60.6	**<.05**
*Treponema pallidum*	292	28.7	111	24.1	181	32.6	**<.05**
Other STI	291	28.6	115	24.9	176	31.7	**<.05**

Statistically significant *P* values (*P* < .05) are shown in bold.

Abbreviations: PrEP, pre-exposure prophylaxis; STI, sexually transmitted infection.

^a^Includes ecstasy, speed, chrystal meth, mephedrone, bath salts, GHB/GBL, ketamine, acid, cocaine, and crack.

The median age of participants at enrollment was 33 years (IQR, 28–39). Participants who were already using PrEP at the start of the study tended to be older, with a median age of 34 years (IQR, 30–40), in contrast to those not using PrEP (median, 31 years [IQR, 27–36]; *P* < .05). The majority of participants identified as gay or homosexual (92.0%) and cisgender men (99.1%), and 67.8% were born in Germany (Table 1). Participants holding a master or doctorate degree were more common among PrEP users as compared with non-PrEP users (40.6% vs 29.9%, *P* < .05).

Among all participants, 81.0% (n = 824) reported engaging in sexual activity with casual sex partners in the 12 months preceding the study entry. Of these, 73.2% (n = 603) had >10 different casual partners, and those using PrEP at the beginning of the study tended to have a higher number of casual sexual partners as compared with non-PrEP users (62.9% vs 54.9%, *P* < .05; Table 1). Of those who engaged in casual sexual encounters, 92.8% (n = 765) disclosed that they had participated in condomless anal sex with a casual partner within the previous 12 months, with slight differences between PrEP users and nonusers (95.8% vs 89.4%, *P* < .05). Of participants reporting sex with casual sex partners (V3, n = 902; V12, n = 743), the proportion having anal sex without condom with a casual sex partner since the last visit decreased significantly from 80.7% (V3) to 76.6% (V12, *P* < .05). There was no difference in the distribution of categories of casual sex partner numbers having anal sex without condom with since the last visit between visits before and after the beginning of the COVID-19 pandemic (*P* = .50). A small proportion (4.4%, n = 45) reported having paid for sex with a male partner within the past year, while 6.1% (n = 62) had been compensated for sexual activities.

Illicit drug use and the use of recreational drugs often associated with sexual contexts (chemsex) ever before study entry were reported by 65.6% and 55.0%, respectively, with no discernible differences based on PrEP use at enrollment (66.6% in non-PrEP users vs 64.7% in PrEP users, *P* = .54; 55.3% vs 54.7%, *P* = .84; Table 1).

### STI Prevalence Over the Entire Study Period

Throughout the entire study period, 71.8% (730/1017) of participants received a diagnosis for at least 1 infection with CT, MG, NG, or TP. More than one-third of all participants had CT (40.4%, 411/1017), MG (38.5%, 392/1017), and NG (36.0%, 366/1017), and 10.1% (103/1017) tested positive for TP. Only 1 infection with *T vaginalis* was diagnosed throughout the study. We therefore excluded *T vaginalis* from further analyses. For HIV, 5 infections were diagnosed over the course of the study, resulting in a period prevalence of 0.5%, with all but 1 in non-PrEP users.

The overall prevalence was most pronounced for anorectal infections (CT, 32.8%; MG, 30.1%; NG, 24.6%); in cases of CT and MG, this was followed by urogenital infection (11.0% each) and oral infection (CT, 7.2%; MG, 8.8%). For NG, oral infections (22.4%) were most frequent, followed by urogenital (2.9%).

**Figure 2.**
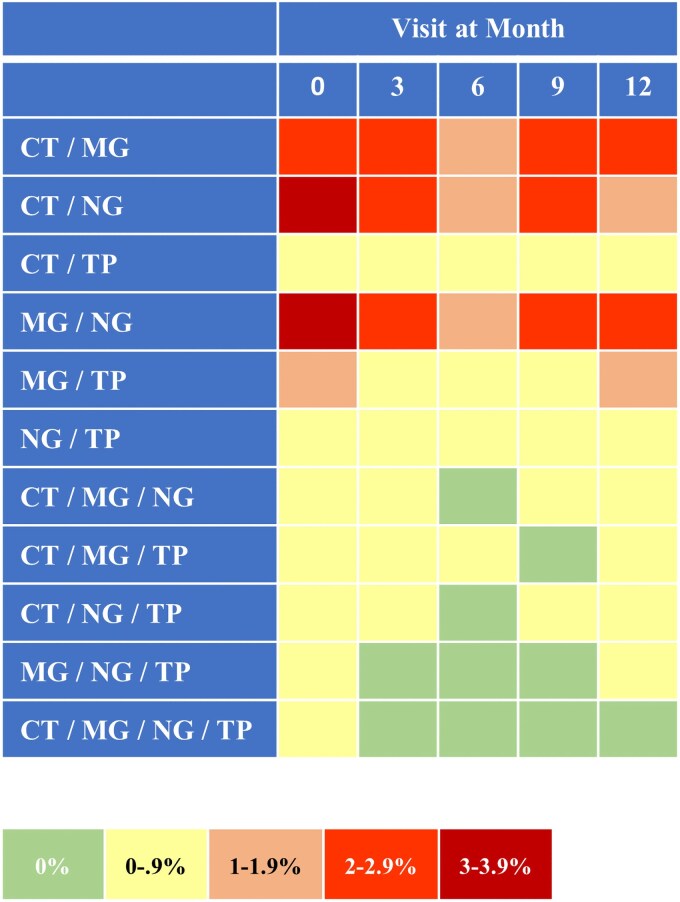
Prevalence of concurrent coinfections at the same study visit with *Chlamydia trachomatis* (CT), *Mycoplasma genitalium* (MG), *Neisseria gonorrhoeae* (NG), and *Treponema pallidum* (TP) by study visit. Absolute numbers of participants by visit: month 0, n = 1017; month 3, n = 1009; month 6, n = 1000; month 9, n = 985; month 12, n = 969.

The most common multiple infections identified during the entire study period were CT/NG (19.8%), CT/MG (18.6%), MG/NG (16.9%), and CT/MG/NG (10.4%). Coinfections diagnosed during the same study visits were most prevalent for CT/NG (10.9%), MG/NG (10.7%), and CT/MG (10.7%; Figure 2).

**Figure 3.**
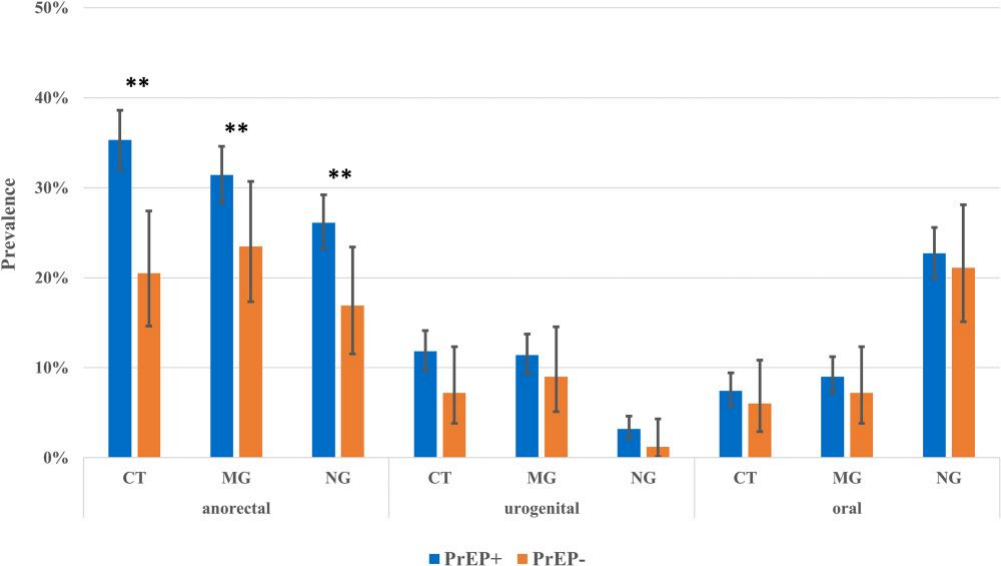
Prevalence of sexually transmitted infections over the entire study period by use of HIV pre-exposure prophylaxis (PrEP; ever over the entire study period) and anatomic site (several participants tested positive for a sexually transmitted infection at multiple anatomic sites). Error bars indicate 95% CI. **P < .05, Mann-Whitney *U* test. CT, *Chlamydia trachomatis*; MG, *Mycoplasma genitalium*; NG, *Neisseria gonorrhoeae*.

The overall STI prevalence was significantly higher in participants who reported PrEP use than in non-PrEP users for CT (43.1% vs 26.5%, *P* < .05) but not for NG (37.3% vs 29.5%, *P* = .06), TP (10.9% vs 6.0%, *P* = .06), and MG (39.5% vs 33.7%, *P* = .16). We found a significantly higher prevalence between participants who used PrEP at least once and non-PrEP users for anorectal infections for CT (35.3% vs 20.5%, *P* < .05), MG (31.4% vs 23.5%, *P* < .05), and NG (26.1% vs 16.9%, *P* < .05; Figure 3).

### STI-Related Symptoms

In 14.4% of all participants positively diagnosed, STI-related symptoms were reported at the visit by the treating physician—most frequently for TP (21.8%), followed by NG (19.4%), CT (17.9%), and MG (12.0%). In the case of CT, symptoms were reported most frequently for oral infections (28.6%), less often for urogenital (20.2%) and anorectal (18.1%). For MG, symptoms were reported with similarly low frequency for urogenital (13.4%), anorectal (12.1%), and oral infections (10.9%). Regarding NG, symptoms were reported most frequently for urogenital infections (31.3%), more seldom for oral (20.6%) and anorectal (18.6%).

### Three-Month STI Incidence

Over the study period, we monitored 995 PY. MG continuously showed the highest 3-month incidence, increasing from 58.8 cases per 100 PY at the month 3 visit (V3) to 81.9 cases at V9 and decreasing to 63.7 cases at V12. For 43.2% of all MG infections diagnosed at V3 to V12 (n = 630), a prior MG diagnosis without MG-related treatment was reported for the preceding visit of the same participant. These diagnoses could also be due to a persistent MG infection that was not treated. If only positive MG test results without a positive test result in 1 or, if applicable, more consecutive previous visits were considered, the incidence of MG showed a similar dynamic on a lower level, increasing from 20.9 cases per 100 PY at V3 to 32.8 cases at V9 and decreasing to 20.6 cases at V12.

**Figure 4.**
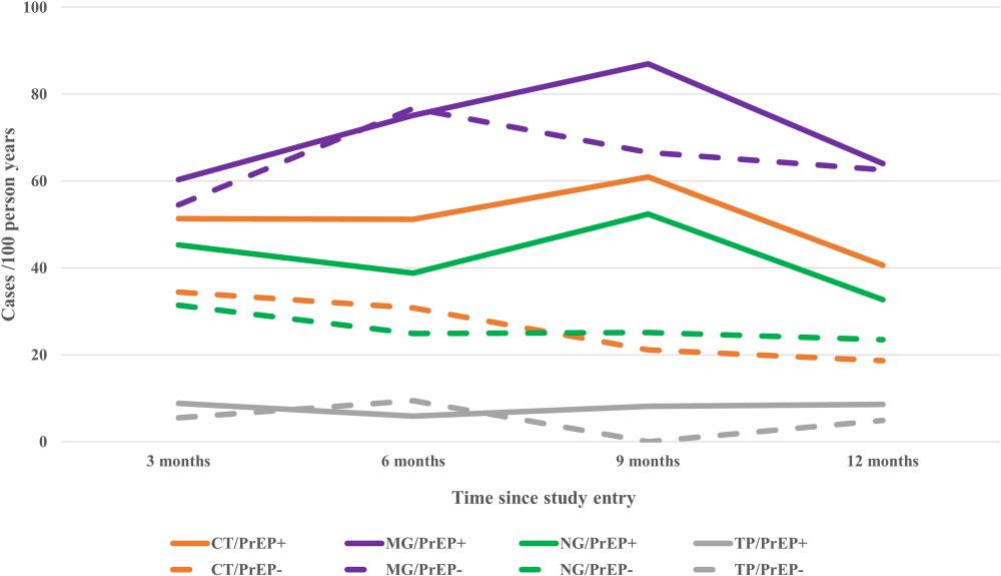
Three-month sexually transmitted infection incidence rates by study visit, pathogen, and use of HIV pre-exposure prophylaxis (PrEP). For MG, all positive test results were counted, including sequential positive MG test results in 1 or more of the preceding visits. CT, *Chlamydia trachomatis*; MG, *Mycoplasma genitalium*; NG, *Neisseria gonorrhoeae*; TP, *Treponema pallidum*.

The 3-month incidence of CT remained relatively stable from V3 to V9, ranging between 46.2 (V6) and 50.7 (V9) cases per 100 PY. For NG, it decreased slightly from 41.6 (V3) to 35.4 (V6) cases per 100 PY and then increased to 45.5 cases (V9). The 3-month incidence of TP remained stable throughout the study period, ranging between 6.1 and 7.9 cases per 100 PY. Regarding HIV, the 3-month incidence was low overall and ranged from 0.7 cases per 100 PY (V3) to 0 (V6), 0.9 (V9), and 0.4 (V12). For CT, MG, and NG, the 3-month incidence among participants reporting PrEP use was consistently higher than those not reporting PrEP use but not for TP (Figure 4; for MG without preceding positive test results, see Supplementary Figure 1).

Over the entire study (between V3 and V12), the 3-month incidence of CT and NG decreased by 23.7% (*P* < .05) and 26.2% (*P* < .05), respectively. Changes for MG and TP were small and not statistically significant (+8.3%, *P* = .48; −2.5%, *P* = .93). Stratified by PrEP use, the 3-month incidence decreased significantly for NG in PrEP users only by 27.8% (*P* < .05; Supplementary Table 1).

When stratified by localization and PrEP use, the 3-month incidence of CT, MG, and NG was higher for PrEP users, specifically for anorectal infections but not for oral or urogenital infections (Supplementary Figure 2, Supplementary Table 1).

Next, we investigated factors associated with acquiring any STI. In the multivariable regression model, the strongest independent associations with the diagnosis of at least 1 STI were observed for participants reporting STI-related symptoms (OR, 1.85; 95% CI, 1.46–2.34), condomless anal sex with >5 casual partners (OR, 1.79; 95% CI, 1.49–2.14), or recreational drug use (OR, 1.76; 95% CI, 1.46–2.12), all since the last study visit (Table 2). Other significant factors were being born outside of Germany (OR, 1.49; 95% CI, 1.19–1.87) and having a moderately or largely increased self-perceived risk of HIV (OR, 1.26 [95% CI, 1.03–1.53]; OR, 1.33 [95% CI, 1.02–1.74], respectively). Despite adjustment by several sexual behavioral factors, PrEP use since last study visit also showed an independent association with being diagnosed with any STI (OR, 1.3; 95% CI, 1.1–1.6).

**Table 2.** Unadjusted and Adjusted Odds Ratios for Factors Associated With Diagnosis of Any Sexually Transmitted Infection Over the Study Period

	Bivariable Analysis			Multivariable Analysis		
	OR	95% CI	*P* Value	OR	95% CI	*P* Value
Age, y						
18–29	1 [Ref]			1 [Ref]		
30–39	0.88	.76–1.01	.07	0.82	.65–1.03	.09
40–49	0.74	.62–.88	**<.05**	0.67	.50–.91	**<.05**
50–55	0.60	.42–.85	**<.05**	0.69	.39–1.24	.22
Place of birth						
Germany	1 [Ref]			1 [Ref]		
Other country	1.49	1.31–1.69	**<.05**	1.49	1.19–1.87	**<.05**
No. of casual sex partners with condomless anal intercourse since last visit						
≤5/no answer	1 [Ref]			1 [Ref]		
>5	2.20	1.94–2.49	**<.05**	1.79	1.49–2.14	**<.05**
PrEP use since last visit						
Yes	1.57	1.37–1.79	**<.05**	1.29	1.05–1.58	**<.05**
No	1 [Ref]			1 [Ref]		
Use of recreational drugs since last visit^a^						
Yes	2.08	1.84–2.35	**<.05**	1.76	1.46–2.12	**<.05**
No/no answer	1 [Ref]			1 [Ref]		
Self-perceived risk of HIV since last study visit						
None/small	1 [Ref]			1 [Ref]		
Moderate	1.22	1.05–1.42	**<.05**	1.26	1.03–1.53	**<.05**
Large/very large	1.61	1.33–1.96	**<.05**	1.33	1.02–1.74	**<.05**
No answer	0.12	.06–.23	**<.05**	1.12	.38–3.29	.84
Acute STI-related symptoms						
Yes	1.72	1.43–2.07	**<.05**	1.85	1.46–2.34	**<.05**
No	1 [Ref]			1 [Ref]		

*Chlamydia trachomatis*, *Mycoplasma genitalium*, *Neisseria gonorrhoeae*, or *Treponema pallidum*. Cases for multivariable analysis: n = 4783. Statistically significant *P* values (*P* < .05) are shown in bold.

Abbreviations: OR, odds ratio; PrEP, pre-exposure prophylaxis; Ref, reference; STI, sexually transmitted infection.

^a^Includes ecstasy, speed, chrystal meth, mephedrone, bath salts, GHB/GBL, ketamine, acid, cocaine, and crack.

## DISCUSSION

In our multicenter prospective cohort study, we examined a population of MSM who were behaviorally vulnerable to HIV and other STIs and eligible for PrEP. Key to our approach was the systematic STI testing across various anatomic sites and treatment, adhering to international guidelines for PrEP services, allowing for insights into the impact of structured STI management on incidence rates among MSM using PrEP or not.

Our findings highlighted a significant STI burden. PrEP users at enrollment reported more casual sexual partners and a history of STIs as compared with nonusers, although behaviors such as condomless anal sex with casual sex partners or recreational drug use, which influence the risk to acquire HIV and other STIs, were similar across groups. Our findings were consistent with those of other studies exploring the relationship between STI rates and PrEP usage [2, 5, 9, 11, 24–27].

Notably, >80% of STIs would have remained undetected without our systematic screening approach, emphasizing the prevalence of asymptomatic infections. Concordant with other studies, asymptomatic anorectal infections were most common [9].

The higher prevalence of engaging in condomless anal sex with casual partners among PrEP users may be the key factor leading to a statistically significant increase in the prevalence of TP and anorectal infections caused by CT, MG, and NG among participants who used PrEP at least once. Indeed, we did not find significant differences in urethral or oral infection supporting the notion that lack of condom use increases risk for anorectal STIs, but this could also be due to a smaller sample size of these groups.

Overall, we found a high incidence of STIs in our study population of MSM eligible for PrEP. The period prevalence of HIV was low, pointing to sufficient knowledge and effective application of HIV-related risk reduction strategies in the study population including PrEP use. We observed a notable reduction in CT and NG incidence over the study period, attributing this to the efficacy of our testing and treatment protocol, potentially augmented by intensified STI prevention counseling. MG infections, however, remained high while considering all MG-positive test results, including sequential positive test results in 1 or more several preceding visits that were, for example, not or insufficiently treated. This highlights (1) challenges in managing these frequently asymptomatic infections; (2) debates about the possibly minor clinical impact of asymptomatic MG infections; (3) the risk of antimicrobial resistance, especially while treating coinfections with other STIs; and (4) the possible harm of frequent antibiotic treatment to the microbiome [15, 16]. In this regard, current clinical guidelines do not support testing of asymptomatic individuals for MG [18]. A distinct proportion of MG infections were consistently not treated in our study.

Regarding the high incidence of CT and NG and the lower incidence of TP, our results were comparable to other studies [4, 5, 9, 11]. As intensified screening for TP was recommended for MSM with increased sexual risk behavior already before PrEP-related screening for CT and NG was introduced in Germany, we did not expect major changes in TP incidence. Our study results differed partly from studies conducted earlier in the scope of PrEP implementation projects that found increasing STI incidence [4, 5], but our findings were in line with those of a large study using real-life clinical data of PrEP users in Australia [11] and another study from Germany [13].

A significant proportion of participants had concurrent infections. Therefore, it appears important for clinicians to be aware of potential coinfections to select appropriate antibiotics to prevent further emergence of antimicrobial resistance. This holds particular significance for asymptomatic infections involving MG and NG [14–16].

Behavioral factors, including condomless anal sex with multiple partners [5, 24, 28, 29] and use of recreational drugs or chemsex, were strongly associated with STI diagnoses and concordant to other studies [30, 31]. Even if an association does not necessarily mean a cause-effect relationship, the results underscore the need for targeted prevention strategies. PrEP use itself had a less direct association with STI risk, probably mediated by these behaviors, as also shown in a study by Hart et al [25].

Potential benefits and costs of STI screening are under debate. Positive effects could be to avoid potential clinical sequelae and a reduction of the population bacterial load and therewith STI transmission, including reducing the risk of acquiring HIV. In contrast, possible damages to the microbiome, high financial costs (especially for 3-month screening intervals), and potential problems with adherence to screening are concerns [16, 19, 21]. Evidence regarding relevant changes of the microbiome due to antibiotic STI treatment is currently lacking. A review showed that 2- to 3-month intervals for STI screening in PrEP users resulted in a reduced STI positivity rate in PrEP users by 75%, as compared with 50% for 4- to 6-month intervals [19]. Our study points to a potential benefit of regular STI screening and treatment in reducing STI burden among MSM, especially for those using PrEP or at higher risk of STIs. The results showed an impact of 3-month STI screening on the incidence of CT and NG over 1 year, but we are not able to make assumptions on effects of less frequent regular STI screening intervals. Similar benefits and harms as for regular and frequent STI screening are under discussion in the context of antibiotic STI prevention with doxycycline (“Doxy-PEP”) as an additional prevention measure, and guidelines differ among countries [32]. The potential interaction among different kinds and frequencies of STI screening and the use of Doxy-PEP currently remains unclear and, therefore, so does the potential benefit of frequent STI screening for Doxy-PEP users.

A limitation of our study was that we could not measure all potential behavioral effects associated with risk of an STI diagnosis. This applies especially to the direct effect of PrEP use that we found in our multivariable model, which might be a sum of additional influencing factors or confounders that we were not able to measure. In addition, our study was conducted at urban MSM-friendly clinics in Germany and may not be generalizable to other settings. A further limitation was that no genetic sequence data for MG infections were available to differentiate persistent or recurrent infections in case of a sequential positive test result in the preceding visit.

## Supplementary Material

ofag205_Supplementary_Data

## Supplementary Data

Supplementary materials are available at *Open Forum Infectious Diseases* online. Consisting of data provided by the authors to benefit the reader, the posted materials are not copyedited and are the sole responsibility of the authors, so questions or comments should be addressed to the corresponding author.
