# Supplementary material for: A Prospective Incidence Study on Sexually Transmitted Infections and HIV in Men Who Have Sex With Men, With or Without Use of Pre-exposure Prophylaxis
Source: Open Forum Infect Dis. 2026 Apr 11;13(5):ofag205. doi: 10.1093/ofid/ofag205 (PMC13123855; doi:10.1093/ofid/ofag205)
Supplement: ofag205_Supplementary_Data [file ofag205_supplementary_data.docx]

Supplementary Materials for Manuscript: *A Prospective Incidence Study on Sexually Transmitted Infections and HIV in Men Who Have Sex with Men with or without Use of Pre-Exposure-Prophylaxis*

# Supplementary table S1: Changes in 3-months incidence of STI over the entire study period (depicted by incidence rate ratios (IRR) between V3 and V12), by pathogen, localization and use of HIV pre-exposure prophylaxis (PrEP)

| **Localization/pathogen/PrEP use** | **Change** | | **p-value** |
| --- | --- | --- | --- |
|  | Difference | IRR |  |
| **Any Localization** |  |  |  |
| CT/PrEP+ | -20.9% | 0.79 | 0.12 |
| MG/PrEP+ | 6.1% | 1.06 | 0.64 |
| NG/PrEP+ | -27.8% | 0.72 | **<0.05** |
| TP/PrEP+ | -2.8% | 0.97 | 0.93 |
| CT/PrEP- | -45.9% | 0.54 | 0.09 |
| MG/PrEP- | 14.9% | 1.15 | 0.56 |
| NG/PrEP- | -25.2% | 0.75 | 0.41 |
| TP/PrEP- | -10.9% | 0.89 | 0.90 |
| **Anorectal** |  |  |  |
| CT/PrEP+ | -11,4% | 0.89 | 0.78 |
| MG/PrEP+ | 2,4% | 1.02 | 0.88 |
| NG/PrEP+ | -19.9% | 0.80 | 0.25 |
| CT/PrEP- | -46.8% | 0.53 | 0.11 |
| MG/PrEP- | 7.4% | 1.07 | 0.79 |
| NG/PrEP- | -16.4% | 0.84 | 0.73 |
| **Urogenital** |  |  |  |
| CT/PrEP+ | -29.7% | 0.70 | 0.24 |
| MG/PrEP+ | 18.8% | 1.19 | 0.54 |
| NG/PrEP+ | 70.2% | 1.70 | 0.42 |
| CT/PrEP- | -27.5% | 0.72 | 0.68 |
| MG/PrEP- | 19.5% | 1.20 | 0.68 |
| NG/PrEP- | - * | -* | 0.46 |
| **Oral** |  |  |  |
| CT/PrEP+ | -10.3% | 0.90 | 0.78 |
| MG/PrEP+ | 6.5% | 1.07 | 0.84 |
| NG/PrEP+ | -37.9% | 0.62 | **<0.05** |
| CT/PrEP- | 81.2% | 1.81 | 0.55 |
| MG/PrEP- | -27.5% | 0.72 | 0.68 |
| NG/PrEP- | -30.8% | 0.69 | 0.36 |
| Abbreviation: IRR: Incidence Rate Ratio  * IRR could not be calculated as incidence at V3 was 0, and division by 0 not possible  Statistically significant p-values (p<0.05) are shown in bold.  Note: for MG, all positive test results were counted, also having sequential positive MG test results in one or more of the preceding visits. | | | |

# Supplementary figure S1: 3-month sexually transmitted infection incidence rates by study visit, by pathogen and use of HIV pre-exposure prophylaxis (PrEP); for MG, only positive test results without positive test result in the preceding visit were counted.

# Supplementary figure S2: 3-month sexually transmitted infection incidence rates by study visit, by pathogen, use of HIV pre-exposure prophylaxis (PrEP), and localization


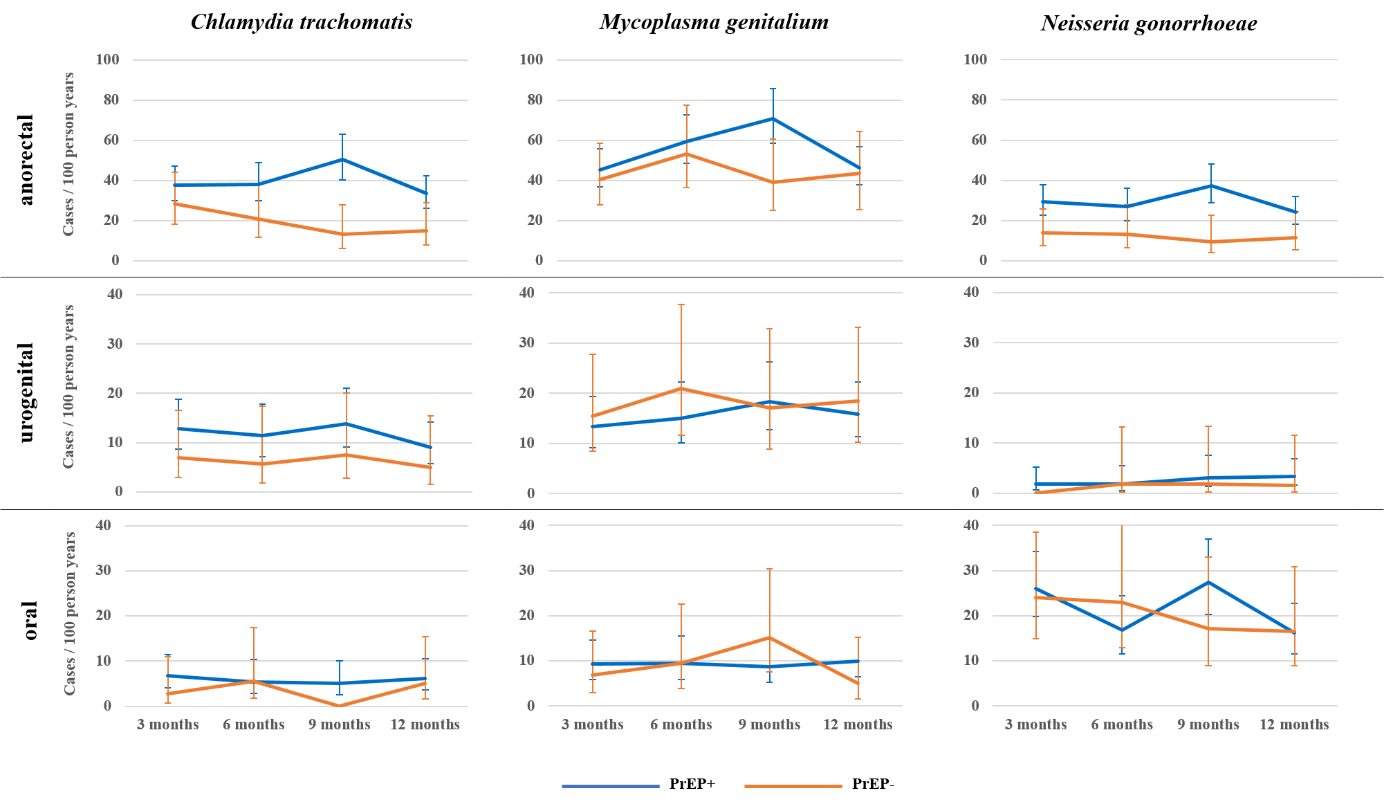


Note: for MG, all positive test results were counted, also having sequential positive MG test results in one or more of the preceding visits.
